# Supplementary material for: β-Neoendorphin Enhances Wound Healing by Promoting Cell Migration in Keratinocyte
Source: Molecules. 2020 Oct 12;25(20):4640. doi: 10.3390/molecules25204640 (PMC7587199; doi:10.3390/molecules25204640)
Supplement: Supplementary file 1 [file molecules-25-04640-s001.pdf]

Figure S1

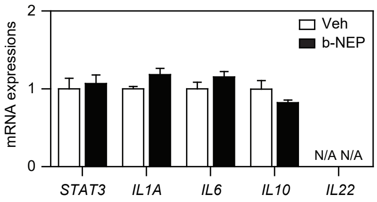

Supplementary figure 1. Effects of β-NEP on inflammatory responses in human keratinocyte.

Q-PCR analysis of inflammatory genes. Expression of indicated genes with or without β-NEP in human keratinocytes. Data represent mean value ± SEM.

Figure S2

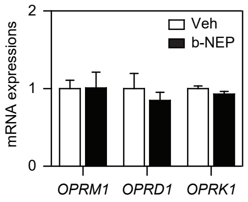

Supplementary figure 2. Expression pattern of opioid receptors after β-NEP treatment in human keratinocyte.

Q-PCR analysis of opioid receptors in human keratinocytes (HaCaT cells). Expression of indicated genes with or without β-NEP treatment for 24 hours.

OPRM1, μ-opioid receptor; *OPRD1*, δ-opioid receptor; and *OPRK1*, κ-opioid receptor. Data represent mean value ± SEM.
